# Are Small GTPases Signal Hubs in Sugar-Mediated Induction of Fructan Biosynthesis?

**DOI:** 10.1371/journal.pone.0006605

**Published:** 2009-08-12

**Authors:** Tita Ritsema, David Brodmann, Sander H. Diks, Carina L. Bos, Vinay Nagaraj, Corné M.J. Pieterse, Thomas Boller, Andres Wiemken, Maikel P. Peppelenbosch

**Affiliations:** 1 Botanisches Institut der Universität Basel, Zurich Basel Plant Science Center, Basel, Switzerland; 2 Plant-Microbe interactions, Institute of Environmental Biology, Utrecht University, Utrecht, The Netherlands; 3 Department of Cell Biology, University Medical Center Groningen, University of Groningen, Groningen, The Netherlands; Gothenburg University, Sweden

## Abstract

External sugar initiates biosynthesis of the reserve carbohydrate fructan, but the molecular processes mediating this response remain obscure. Previously it was shown that a phosphatase and a general kinase inhibitor hamper fructan accumulation. We use various phosphorylation inhibitors both in barley and in Arabidopsis and show that the expression of fructan biosynthetic genes is dependent on PP2A and different kinases such as Tyr-kinases and PI3-kinases. To further characterize the phosphorylation events involved, comprehensive analysis of kinase activities in the cell was performed using a PepChip, an array of >1000 kinase consensus substrate peptide substrates spotted on a chip. Comparison of kinase activities in sugar-stimulated and mock(sorbitol)-treated Arabidopsis demonstrates the altered phosphorylation of many consensus substrates and documents the differences in plant kinase activity upon sucrose feeding. The different phosphorylation profiles obtained are consistent with sugar-mediated alterations in Tyr phosphorylation, cell cycling, and phosphoinositide signaling, and indicate cytoskeletal rearrangements. The results lead us to infer a central role for small GTPases in sugar signaling.

## Introduction

Sucrose is the central transport sugar in plants. It is the ultimate end product of photosynthesis and is exported from source cells in leaves. After transport it is taken up by sink cells in for example the root. A surplus of sugar is usually stored for times when less light is available. Sugar can be stored as reserve carbohydrate for example in the form of starch or fructan. In plants that use fructan as reserve carbohydrate, e.g. grasses, light and high sucrose levels induce fructan production [for a review see 1]. One of the more established mechanisms by which these signals increase fructan content is via the induction of transcription of genes encoding fructosyltransferases, the enzymes that produce fructan [2]–[7] and biotechnological modification of common crops to stimulate and modulate fructan synthesis has become an important activity [8], [8]–[12]. Despite an intense research effort, the molecular details by which excess sugar provokes transcription of fructosyltransferases remain largely obscure.

Reversible protein phosphorylation is a key mechanism for intracellular signal transduction in eukaryotic cells. A general inhibitor of protein kinases and a phosphatase inhibitor have been reported to inhibit the induction of fructan synthesis in wheat by sucrose [13]. Thus, reversible protein phosphorylation may well be important in the signal-transduction leading from increased sugar availability to the induction of fructan synthesis. However, little is known about sucrose signaling and associated glucose and/or fructose signalling in general, let alone the elements that are leading to fructosyltransferase induction. Pontis and collegues have provided evidence that phosphatases and kinases are involved in sugar-mediated fructan induction [14], [15].

Genetic studies suggest differences between kinases in animal cells and plant cells. Plants harbor histidine and aspartate kinases as part of the two-component signaling system. This system was first discovered in prokaryotes, but has not been found in animals so far. In animals, two different types of protein kinases are distinguished; kinases that phosphorylate serine or threonine residues and kinases that phosphorylate tyrosine residues. In plants Ser/Thr-kinases are also abundant and implicated in many signaling events, but classical Tyr-kinases are less well known. In the last years, evidence of a variety of tyrosine phosphorylation events in plants is quickly accumulating to such an extend as being hardly controversial anymore [16]–[20], in spite of the absence of classical tyrosine kinases in genomes of plants. It is suggested that dual-specificity kinases that have a relatively high tyrosine phosphorylating activity are responsible for the observed Tyr-phosphorylation activities in plants [21]–[23]

Recently we showed that with regard to kinase substrates, there is little difference between plants, animals, fungi, and yeast [24]. Despite the obvious differences in kinase structures, animal and plant extracts phosphorylate more or less the same set of peptide substrates. Importantly, this opens the theoretical possibility to transplant vertebrate substrate-based tools for assessing kinase activity to plant systems. In a recent study we demonstrated the usefulness of peptide arrays exhibiting a variety of kinase peptide consensus substrates for assessing changes in kinase activity in *Arabidopsis thaliana* upon pathogen infection [25].

The above-mentioned considerations prompted us to investigate the possible role of phosphorylation in sugar responses in plants. To this end, the promoter of a barley fructosyltransferase, was cloned [7]. As this promoter was reported to have a SURE (sugar responsive) element, we assumed that a construct containing this promoter fused to GUS would represent a useful tool for studying sugar signaling *in planta* and this was confirmed by in vivo experiments using transgenic plants. Employing these transgenic plants we show that different classes of kinases and phosphatases are indeed essential for appropriate induction of fructan synthesizing enzymes. Accordingly, using peptide arrays we were able to demonstrate altered phosphorylation of a set of peptide kinase substrates following sugar feeding and the information obtained was employed to construct a provisional signal transduction scheme of sugar responses in Arabidopsis. The results are consistent with sugar-mediated alterations in Tyr phosphorylation, cell cycling, and phosphoinositide signaling, and cytoskeletal reorganization. Furthermore, the results lead us to infer a central role for small GTPases in sugar signaling.

## Results and Discussion

### Induction of 6-SFT in Barley is dependent on various Phosphatase and Kinase Activities

It was shown that inhibition of phosphatase activity by okadaic acid reduces the induction of fructan synthesis in wheat upon sucrose feeding [26]. To corroborate involvement of phosphatase activity also for the induction of fructan synthesizing enzymes *per se*, we tested the ability of the phosphatase inhibitor okadaic acid for its ability to reduce expression of the fructosyltransferase gene *6-sft* upon sugar feeding. Okadaic acid is a known inhibitor of protein phosphatase 2A (PP2A) and at higher concentrations also of protein phosphatase 1; in contrast protein phosphatase 2C is not sensitive to okadaic acid.

**Figure 1:**
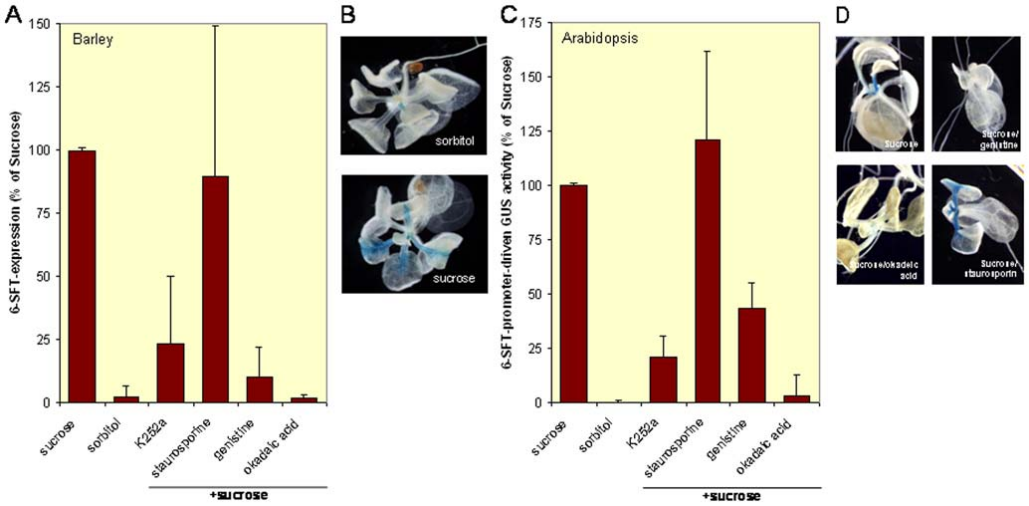
Reversible phosphorylation drives *in planta* induction of the *6-sft* promoter. A. Using qPCR the amount of 6-sft mRNA in barley was determined. As compared to 100 mM sorbitol (osmotic control), treating leaves with 100 mM sucrose substantially induced 6-sft transcripts. This induction was sensitive to 0.1 µM okaideic acid (a S/T phosphatase inhibitor), 0.2 µM K252a ( a broad spectrum kinase inhibitor), and 2 µM genistein (a receptor tyrosine kinase inhibitor) but not to 2 µM staurosporin (a Calcium/calmodulin dependent protein kinase inhibitor). B. To study sugar signal transduction in *A. thaliana*, this plant was transformed with a construct in which the GUS reporter was driven by the barley *6-sft* promoter. Without the addition of external sugars, GUS expression is seen mainly in the apex and in young leaves, disappearing in older leaves. The little induction of GUS activity that was seen following overnight treatment of *A. thaliana* seedlings with a solvent osmotic control (100 mM sorbitol) was similar to non-treated plants, the strong induction of GUS activity as seen following overnight treatment of *A. thaliana* seedlings with 100 mM sucrose is clearly visible. C. Quantification of the GUS activity after addition of various kinase inhibitors and a phosphatase inhibitor on sucrose-mediated GUS induction in *A. thaliana*. D. Examples of plants treated with kinase inhibitors or a phosphatase inhibitor.

We compared expression in sorbitol- and sucrose-fed barley leaves, and we used the combination of sucrose and okadaic acid. Expression in barley was measured using real-time qPCR. Expression of the fructosyltransferase was, as expected, enhanced after sugar application as compared to a sorbitol control (p≪0.01). Upon okadaic acid application sucrose could not enhance 6-SFT expression (Figure 1A; p≪0.01).

The evidence for a possible role of protein phosphorylation in sugar responses was further strengthened in experiments in which various kinase inhibitors were directly tested for their capacity to inhibit the induction of the fructosyltransferase promoter following sugar feeding. The general kinase inhibitor K252a was tested for sucrose-induced *6-sft* fructosyltransferase transcription in barley. qPCR analysis showed that the application of the inhibitor K252a reduces induction of *6-sft* transcription by sucrose in barley to 20% (Figure 1A; p<0.05)), indicating that kinase activity is necessary for *6-sft* expression.

To obtain insight into which classes of kinases are involved in this transactivation, inhibitors of specific classes of kinases were tested. Staurosporine is a general inhibitor of AGC protein kinases at high concentrations, but at lower concentrations it becomes more selective for protein kinase C (PKC) and Calcium/calmodulin depenent protein kinases (CDPK) [27]–[29]. The presence of PKC is not unambiguously proven in plants, but CDPKs are present in large amounts [30]. In our experiments, staurosporine was not able to inhibit sugar-mediated induction of the fructosyltransferase promotor in barley at the low concentrations used (Figure 1A), suggesting that CDPKs are not important players in *6-sft* induction. Martinez-Noel *et al*. [31] recently reported induction of the transcription of a CDPK in wheat upon sugar feeding. This points to a role for CDPKs in sugar signaling, our results however suggest that this expression is not important for the induction of fructosyltransferases, but serves another purpose. Interestingly, in agreement with a previous reported role for calcium in fructosyltransferase induction [32], in our experiments Quin2-AM, a calcium-signaling inhibitor, was able to inhibit sugar induction of GUS activity with on average 1/3, although with high interexperimental variability (data not shown).

Canonical Tyr-kinases are not obviously present in plants as deduced from their primary sequence, but Tyr-kinase activity is evidently present and of physiological importance in plants [24], [33]–[35]. In apparent agreement, the receptor Tyr-kinase inhibitor genistein has been proven to be a useful tool to study Tyr-kinase involvement in plants [17], [36]. We show that genistein is able to inhibit sucrose-dependent *6-sft* gene transcription as assayed by qPCR of *6-sft* in barley (Figure 1A; p<0.01). Thus Tyr-kinase enzymatic activity seems essential for sugar-dependent fructosyltransferase gene transcription.

### Studying fructosyltransferase induction in *Arabidopsis thaliana* reveals that reversible phosphorylation is important for responses to sucrose feeding in divergent plant species

*Arabidopsis thaliana* Col-0 was transformed with 1.5 kb of the promoter of the barley fructosyltransferase 6-SFT [7] in front of a β-glucoronidase (GUS) reporter gene. It was shown before that the GUS behind this promoter sequence could be induced by sugar in barley leaves [7]. Three independently transformed Arabidopsis lines were checked for GUS expression during growth, without the addition of external sugars. GUS expression could sometimes be seen in the apex of young leaves (data not shown).

Upon sucrose feeding GUS-expression is enhanced, especially in the petiole veins expression was well visible. As an osmotic control for sucrose we used the non-metabolizable sugar sorbitol. Sorbitol did not alter GUS expression compared to water and DMSO control treatment (Figure 1B and data not shown). Quantification of the GUS expression using MUG showed indeed an increase in GUS activity upon sucrose feeding (Figure 1C; p≪0.01). We conclude that the promoter-GUS construct in Arabidopsis can be used to study sugar-dependent transactivation the barley fructosyltransferase 6-SFT promoter.

Since many of the inhibitors are dissolved in DMSO we tested the effect of DMSO on its own and in combination with sucrose on GUS activity. DMSO did not induce GUS and did not inhibit sucrose-induced GUS (data not shown).

To corroborate the usefulness of the promoter-GUS construct in Arabidopsis we tested the same inhibitors as described above with barley and studied the impact on sucrose-induced GUS expression. Also in Arabidopsis okadaic acid reduced sucrose-induced GUS expression from the fructosyltransferase promoter to background levels (Figure 1C,D; p<0.01), without apparent interference with plant viability (not shown). Hence, inhibition of PP2A interferes with sugar-dependent gene expression in divergent plant species.

The general kinase inhibitor K252a that diminished sucrose-induced *6-sft* fructosyltransferase transcription in barley was tested in the same Arabidopsis lines. GUS activity reduced to 21% of the sucrose-induced GUS activity when K252a and sucrose we present together (Figure 1C; p<0.01), quite comparable to the reduction we observed in barley. Thus protein kinase activity is involved in the sugar-mediated gene expression as assayed by transactivation of the fructosyl transferase promotor.

As in barley, staurosporine at fairly low concentrations was not able to inhibit sugar-mediated induction of the fructosyltransferase promotor in Arabidopsis (Figure 1C). This suggests that CDPKs are not important for the signal transduction cascade from sugar to the induction of fructan biosynthesis, leaving room for other kinases to be involved. The Tyr-kinase inhibitor genistein was able to inhibit sugar-driven *6-sft* expression in barley. We observed that genistein is able to inhibit sugar-dependent gene transcription also in transgenic Arabidopsis (Figure 1C,D; p<0.05). Thus Tyr-kinase enzymatic activity seems essential for sugar-dependent gene transcription throughout the plant kingdom.

### A prerequisite for protein phosphatase 2A enzymatic activity for sugar-dependent fructosyltransferase induction

**Figure 2:**
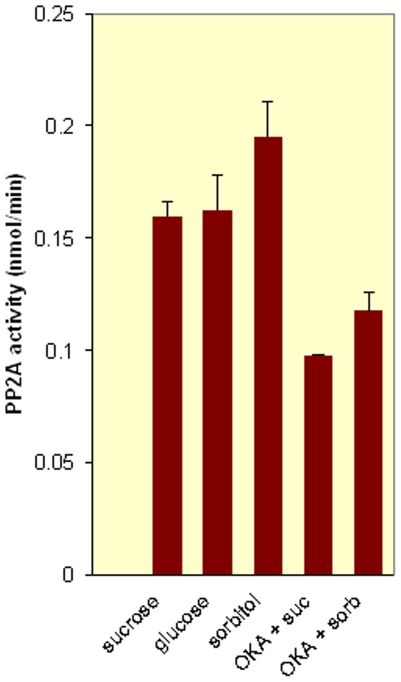
Sugar signaling does not increase PP2A activity. PP2A activity was measured by an imunoprecipitation followed by a malachite green assay as described in the methods. The PP2A-specific component was isolated by comparing data with *in vivo* okadeic acid-treated samples (0.1 µM). The results show that 100 mM sucrose treatment does not increase PP2A enzymatic activity, but do confirm that okadeic acid is an efficient inhibitor of PP2A activity.

In cell cultures okadaic acid concentrations up to 1 µM did selectively affect PP2A [37]. In our initial experiments we used 0.5 µM okadaic acid with whole plants and could repress the sugar-induced expression of fructosyltransferase completely. Also when the okadaic acid concentration was lowered to 0.1 µM, complete inhibition was observed (residual activity 5%±14%). Therefore we reasoned that PP2A was probably active in sugar signaling and decided to directly test the activity of PP2A after sugar feeding. We used immune precipitation of PP2A from lysates of Arabidopsis plants that were incubated with sugars for 1 hour and detected PP2A activity using the Malachite green assay (see methods). Arabidopsis shows a considerable basal PP2A activity that was to our surprise not further increased upon sucrose feeding, nor altered by sorbitol (Figure 2). However, in plants to which okadaic acid was applied PP2A activity was inhibited, both in sucrose and sorbitol-fed plants (p<0.01). This indicates that the constitutive levels of PP2A activity are sufficient for sugar-mediated induction of the fructosyltransferase promotor and that PP2A activty does not need to be enhanced for sugar-mediated induction of fructan biosynthesis.

This phenomenon that the activity of an essential component of the signal transduction cascade is not enhanced during signaling, whereas at the same time being essential for signaling, is found before in e.g. phorbol-mediated activation of PKC for which basal calcium levels are sufficient. Decreasing cytosolic calcium levels below a certain level renders activation of PKC impossible [38].

### Kinome analysis shows differentially phosphorylated peptides after sugar feeding

**Figure 3:**
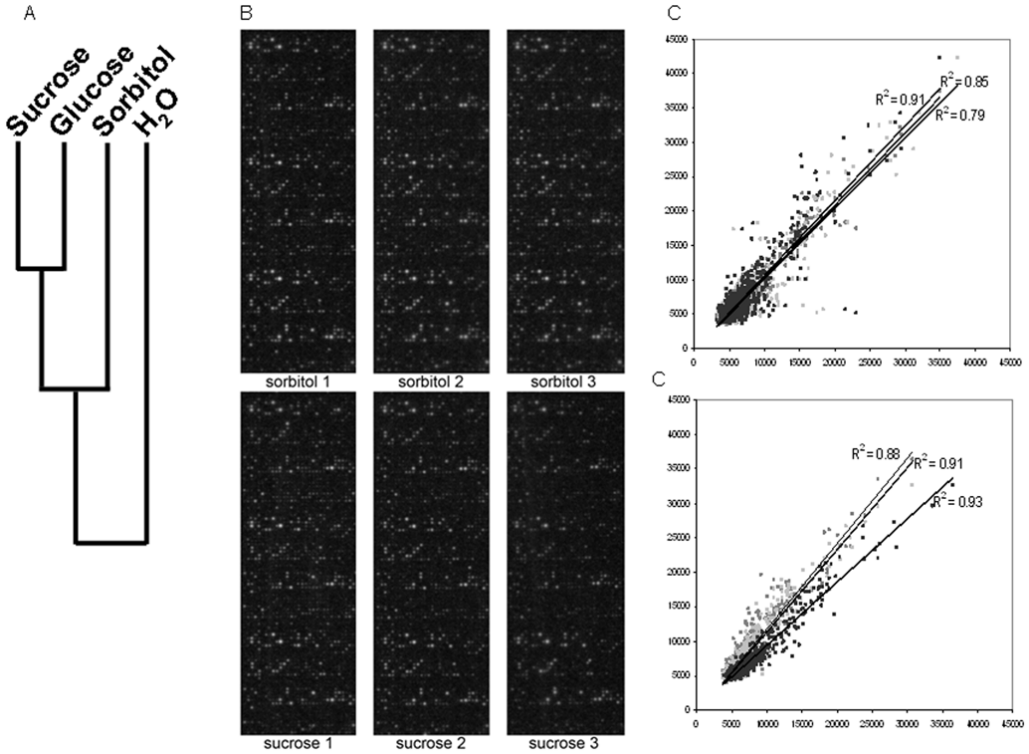
Generation of phosphorylatation profiles. Lysates of plants either treated with water,100 mM sorbitol, 100 mM Glucose or 100 mM sucrose were incubated with peptide arrays containing 1176 peptide substrates (A; selected for their potential to provide broad coverage of cellular phosphorylation) or 1076 peptide substrates (B–D; selected for their importance in mammalian signal transduction) in triplo (three sets of substrates above each other). A. Cluster analysis according to Johnson of the profiles obtained suggest that sucrose and glucose signaling is highly similar and distinct from osmotic control or incubations with water. B. Original scans. C. Correlation plots of the technical replicates for the sample sorbitol 1 show the high reproducibility of the results obtained. D. Correlation plots of the biological replicates for the sucrose-treated samples show the high reproducibility of the results obtained.

Recently, we demonstrated the usefulness of peptide arrays to generate descriptions of changes in global phosphorylation in Arabidopsis following infection with an avirulent strain of the bacterial pathogen *Pseudomonas syringae* pv. *tomato* [25]. Furthermore, in another study we demonstrated that the set of substrates phosphorylated by plant cell extracts is almost identical to that phosphorylated by animal cell extracts, demonstrating that arrays consisting of animal consensus sequences are also useful for studying plant cell phosphorylation [24]. We were interested to see whether pattern of array phosphorylation would reveal relations between the reactions of Arabidopsis to various treatments, including a treatment with Sucrose, Glucose, Sorbitol and the water vehicle. To this end we employed chips consisted of 1152 different nonapeptides, covering the majority substrate peptides available through Phosphobase (version 2.0). and due to their substrate diversity give good approximation as to cellular reactions to different treatments [8] and calculated the spearman correlation between the array results obtained and subsequently clustered the results according to Johnston. The results of the cluster analysis are shown in figure 3. The results showed that Sucrose and Glucose elicit highly similar kinase profiles and thus for practival purposes may be considered equal (henceforth collectively called sugar signalling [which would thus constitute signalling in response to glucose, fructose and sucrose] in this text). Sorbitol was moderaltely different from the results obtained from Sucrose/Glucose pair. Treatment with water alone, however, was markedly different, demonstrating that is not an approppiate control and that the sorbitol osmotic control must be employed to make meaning full stratements as to the effects of sugar signalling on cellular phosphorylation patterns and experiments were designed accordingly. Furthermore, these results show that the responses elicited by sucrose and glucose are highly similar and thus that our results represent a common sugar response rather as a narrow sucrose-specific phenomenon. Using a peptide array containing 1024 different kinase consensus sequences, selected for their importance in mammalian signal transduction, we saw efficient phosphorylation of arrays by plant extracts (Figure 3b). The phosphorylation of these arrays was apparently specific as both the intra-experimental variance (Pearson's correlation product moment r = 0.923±0.027) and inter-experimental variance (r = 0.961±0.012) in peptide substrate phosphorylation was very low (Figure 3c) and the multitude of phosphorylation events detected in this mammalian array seems to confirm the notion that kinase substrates much more then the kinase enzymes themselves have been conserved in eukaryotic evolution [16]. Subsequently, we analyzed the effect of sucrose treatment on the capacity of Arabidopsis extracts to phosphorylate these peptide arrays. As expected, phosphorylation of most peptides was not changed by the treatment, but 43 peptides were statistically significantly differentially phosphorylated following sucrose feeding. A list of the peptides involved is given in table 1, whereas a complete overview of the results obtained for all substrate peptides can be found in the supplementary data (table S1). Phosphorylation regulates a multitude of physiological functions, many of which are not expected to be influenced by altered sugar levels, therefore the limited subset of differentially phosphorylated peptides are probably very specific kinase targets involved sugar-mediated signaling and indicate sugar-specific changes in cellular kinase activities.

**Table 1:** Peptide substrates displaying statistically significant altered phosphorylation when peptide arrays are incubated with lysates of sucrose-treated plants as compared to lysates from sorbitol-treated plants. Peptides were selected for their importance in human signal transduction and represent human sequences. The results show the results of *A. thaliana* treated for 1 hour with either sucrose or the inactive osmotic control, both at a concentration of 100 mM. Subsequently, cells were lysed and analysed for their capacity to in vitro phosphorylate peptide arrays (for details, see main text).

PEPTIDE ON CHIP	tTEST	P UP/DOWN	KINASE	PROTEIN	REMARK
**RERLKSTNDKG**	0,004	UP	AKT, S6K, CK2	Estrogen receptor, alpha	PKB
**RKRSTSLNERP**	0,030	DOWN	AKT1	Tuberin	PKB
**RKRRWSKPESR**	0,046	DOWN	ATK1	Ataxin 1	PKB
**TVKENTKDELK**	0,042	UP	BCR	AF6	
**QLGPPSPVKMP**	0,015	UP	cdc2	Pituitary tumor-transforming protein 1	
**LRQLRSPRRKQ**	0,001	DOWN	cdc2	Ras-like protein Rab4	
**GDKKETPPRPR**	0,011	UP	cdk5	MEK1	
**MSTESMIRD**	0,039	UP	CK1	TNF alpha	
**TTTKPSLSGKG**	0,002	UP	CK1, GSK3beta	Beta-catenin	2 phosphorylation sites in peptide
**TTGGESKDELE**	0,009	UP	CK2	Occludin	
**IRYIESLQELL**	0,023	UP	CK2	Myogenic factor 5	
**NRSFLSLKHTP**	0,004	UP	GSK3beta	Notch2	
**PVKRTSPLQTP**	0,039	DOWN	MAPK (JNK), PKC	Bcl 2	PKC during G2/M for anti-apoptosis
**TIDPKSPQSPE**	0,035	DOWN	MAPK (p38)	Solute carrier family 9, isoform A1	Na/H-antiporter
**IGEGTYGVVYK**	0,039	DOWN	Myt, WEE	CDC2	completely conserved in arabidopsis
**VPKRKSLVGTP**	0,044	UP	PAK6	p21 activated protein kinase 6 (PAK6)	auto-P
**GDRTSTFKGTP**	0,047	UP	PDK1	PKN	PKC-like kinase
**KERRFSRSDQL**	0,012	UP	PKA	WT1	Zinc-finger
**FTRRKSVKKEK**	0,021	UP	PKA	PKA	auto-P
**EKRKNSILNPI**	0,028	DOWN	PKA, PKC	CFTR	ABC transporter, multi-drug resistance protein, pS700 by PKA
**LTRIPSKKKYK**	0,025	UP	PKC	PEA15	death effector domain (DED)-containing protein
**LSPSPSSRVTV**	0,039	DOWN	PKC	Lamin B1	
**KRQYGTISHGI**	0,023	DOWN	PKC	Amyloid precursor like protein 2	
**KFRTPSFLKKN**	0,043	DOWN	PKC	Adducin 3	cytoskeleton
**ESLESTRRILG**	0,007	UP	PKC	SNAP23	vesicle transport, 2 residues phosphorylated by PKC
**ESRSGSNRRER**	0,028	UP	PKC	WIP	Wiskott-Aldrich syndrome protein (WASP) interacting protein
**RKRHNSISEKK**	0,023	DOWN	PKG	Phospholipase C, beta 3	produces DAG and IP3
**SSYRRTFGGKP**	0,023	UP	ROCK	Desmin	Rho-associated kinase ROCK
**EPKRRSKRLSK**	0,043	DOWN	S6K, PKA, PKC	HMG14	
**KDEGSYTLEEP**	0,024	UP	Tyr-K	Syndecan 3	possibly sugar-dependent (lectin domain)
**TDEDIYLLGKK**	0,022	UP	Tyr-K	Sialyltransferase 1	
**SKEPQYQPGDQ**	0,030	UP	Tyr-K Csk	Fgr	Src2
**GGDDIYEDIIK**	0,020	UP	Tyr-K EGFR	VAV2	GEF of Rho-family GTPase
**KENPEYLGLDV**	0,031	DOWN	Tyr-K EGFR	ErbB2	Tyr-K receptor, auto-P
**DGKEIYNTIRR**	0,005	UP	Tyr-K EGFR, Tyr-K Lck	RasGAP	GAP1 (GTPase-activating proteins)
**IPSVPYKPFKK**	0,035	UP	Tyr-K Hck (Src-subfamily)	Guanine nucleotide releasing factor 2	GEF of Ras-like GTPases
**EQRNVYKDYRQ**	0,013	UP	Tyr-K Src	Dynamin 1	vesicular trafficking
**FTNPVYKTLYM**	0,044	UP	Tyr-K Src	Low density lipoprotein receptor-related protein 1	endocytosis
**KDENYYKKQTH**	0,011	UP	Tyr-K SYK	SYK	auto-P, SYK activates VAV
**RQGKDYVGKIP**	0,042	UP	Tyr-K VEGFR2	VEGF receptor 2	auto-P
**KKYMETVKLLD**	0,015	UP	VRK	Vaccinia related kinase 1	auto-P
**MHRRHTDPVQL**	0,046	UP		GRID	GRB2, Tyr-K signaling, Ras protein signal transduction
**SLFDRTPTGEM**	0,012	UP		Myosin light chain 1	

### Changes in tyrosine phosphorylation accompany sugar signaling

Of the 43 peptides of which the phosphorylation is significantly altered following sucrose treatment, 12 peptides are annotated to act as consensus substrates for tyrosine kinases. From the plant genomic sequences available hitherto no obvious classical tyrosine kinases have emerged and of the significant 12 peptides, 7 also contain Ser or Thr residues and may thus, at least theoretically, act as substrates for other Ser/Thr kinases and not represent an effect of sugar feeding on tyr-kinase activity. However, 5 peptides represent changes in tyrosine-specific phosphorylation, in apparent agreement with the sensitivity of sugar-dependent gene expression to the Tyr-kinase inhibitor genistein as described above. Evidence for tyrosine kinase activity in plants has been rapidly increasing in recent years. Barizza *et al.* [39] report many phosphorylated tyrosine residues in plants. In addition, Tyr-phosphrylation is involved in stomatal opening [40]. A possible source of this tyrosine kinase activity are the dual specificity kinases, of which several have now been cloned [41]. Rudrabhatla *et al*. did a genome-wide screen for Arabidopsis Tyr kinases and found that all 57 potential Tyr-kinase also harbor motifs for Ser/Thr-kinases. This indicates that all 57 potential Tyr-kinases are dual specificity kinases which are named STY-kinases [42]. They have been reported to show high Tyr (auto)phosphorylation compared to their activity on Ser and Thr. [43]–[45]. Miranda-Saavedra and Barton [46] also did a search on Tyr kinases in Arabidopsis. They find 2 Tyr-kinases and 776 putative Tyr-like kinases. Most significantly, Nittyla et al. employing Mass Spec identify several phosphotyrosine residues whose levels change upon sucrose stimulation of Arabidopsis seedlings [47]. Taken together, more the evidence that Tyr-kinases are present in plants and are involved in sugar signaling is.compelling.

One of Tyr-phosphorylating peptides (IGEGTYGVVYK) is the WEE/MYT consensus in CDC2. The consensus peptides is completely conserved in the Arabidopsis CDC2, which is called CDKA and it was shown that this CDKA phosphorylation by WEE is also found in Arabidopsis [48]. The functional consequence of the decreased CDKA phosphorylation is almost certainly stimulation of the cell cycle as it is known that WEE phosphorylation of CDC2 is conserved in plants and WEE-mediated phosphorylation of CDC2 inhibits the cell cycle and fits well with established data showing CDC2-dependent induction of the cell cycle following sucrose stimulation of A. Thaliana [49], [50]. Thus sugar appears to stimulate cell division. This regulation would come on top of earlier noticed regulation of the cell cycle by sugar at the level of cyclins [51].

### Evidence for a role of a small GTPase in sugar-dependent signalling

Among the significant changes in tyrosine-containing peptide substrates, the presence of increased tyrosine phosphorylation of three motifs implicated in the activation of small GTPases is striking (GGDDIYEDIIK, IPSVPYKPFKK, DGKEIYNTIRR, see table 1). This suggests that changes in tyrosine phosphorylation mediate the observed effects on sugar-dependent gene transcription via stimulation of (a) small GTPase(s). Small GTPases of the Ras superfamily are reported in plants [for reviews see 52,53]. These small GTPases regulate membrane trafficking, cytoskeletal reorganization, proteasome-mediated proteolysis, cell division and differentiation etc. They are involved in stress, defense, and hormone signaling and presumed to be hubs for signal integration [54], [55]. Small GTPases are represented by 93 genes in Arabidopsis [56]. Of these the ROP (Rho proteins of plants) GTPases are the best studied. They consist of 11 genes in Arabidopsis [57]. In addition to these GTPases, also their regulating factors, GEFs (guanidine exchange factors) and GAPs (GTPase activator proteins), are present in Arabidopsis [58], [59].

Phosphorylation of consensus peptides present in two GEFs (GGDDIYEDIIK, IPSVPYKPFKK) and one GAP (DGKEIYNTIRR) is up after sugar treatment (Table 1). GEFs activate GTPases and phosphorylation of GEFs increases their activity. In contrast, GAPs, which negatively regulate GTPases, are inhibited upon phosphorylation, resulting in the activation of small GTPases following this GAP phosphorylation. Taken together, both GEFs and GAPs seem to be regulated after sugar treatment in such a way that small GTPases are activated. Additional evidence for this conclusion comes from the upregulated phosphorylation of the ROCK and PAK consensus sites (SSYRRTFGGKP and VPKRKSLVGTP, resp.), which are targets of Rho superfamily of GTPases. This activation of small GTPase-dependent kinases after sucrose treatment is an additional proof for small GTPases to be central in sugar signaling. In mammalian signal transduction field use of the C3 toxin is generally considered sufficient to make a statement as the requirement of Rho proteins (the mammalian homologues of ROPs) [60]. At a relatively low concentration of C3 we find inhibition of sugar signalling. In addition we find that two direct Rho family effector peptides are also effected in addition to plethora of peptides indirectly reading out Rho signalling. Upstream we find peptides whose phosphorylation is commonly associated with GEF activation and GAP inactivation. Together these data infer us to propose regulation of small GTPases in sugar signalling both through GEFs and GAPs, but until more precise biochemical assays are developed for plant systems other possibilities should be kept in mind.

### Apparent sugar-induced regulation of additional signal-transduction elements

Phosphoinositide metabolism is intimately associated with activation small GTPases of the Rho family, depending on the exact experimental system acting either upstream or downstream of these GTPases. If such small GTPases are involved in sugar signaling, one would expect to see evidence of phosphoinositide metabolism on the protein array. In apparent agreement we detected significant upregulation of a PDK1 (a strickly phosphoinositide regulated kinase) consensus substrate (GDRTSTFKGTP) following sucrose treatment. Thus, sugar signaling seems associated with up regulation of phopshoinositide metabolism, providing further evidence for activation of small GTPases by sugar.

Four of the differentially regulated substrates constitute consensus peptides for Casein Kinases (CKs). Genomic analysis of the Arabidopsis genome suggests that multiple CKs are present, as various catalytic and regulatory subunits are present in the genome (The Arabidopsis Information Resource (TAIR); http://www.arabidopsis.org/). CKI and CKII are pleiotropic in their function and implicated in general processes such as development, cell cycle progression, chromatin remodeling, and circadian rhythm [61]–[64]. Interestingly, e.g. in Wnt signaling in animals, increased CK enzymatic activity is reported to cooperate with small GTPase-dependent signaling [65], [66], hence co-regulation of CK and small GTPase-dependent kinase activities seems a recurrent theme in eukaryotic biology.

The most prominent effect of Rho superfamily GTPase signaling in general, also in plants, and cooperative CK/GTPase signaling in particular, is a reorganization of the actin cytoskeleton [67–69]. Interestingly, the list of substrates whose phosphorylation is increased after sucrose stimulation includes substrates for Src and its family members Lck and Hck, which in animal systems are well-described to mediate cytoskeletal rearrangement following stimulation of Rho-like small GTPases [70]. In our kinome arrays substrate peptides present in a WASP-interacting protein (WIP) (ESRSGSNRRER), and desmin (SSYRRTFGGAP), two downstream effectors of small GTPases that mediate effects to the actin cytoskeleton [71], show differential phosphorylation. Although the exact orthologues for these substrates in Arabidopsis are not immediately obvious, these results do provide strong support for the notion that activation of small GTPases following sugar stimulation constitutes a biochemically important event.

Conspicuously absent from our analysis is SnRK1, a kinase implicated in sugar sensing in Arabidopsis [72–74]. SnRK1 is the closest homologue of the SNF1/AMPK, in yeast and mammals important regulators of nutrient signaling [72,75]. Only two AMPK consensus peptides are present on the PepChip, and they might not be phosphorylated by the SnRK1 orthologue.

### Inhibitors link Rho-family GTPases and PI3 kinases to fructosyltransferase promoter induction by sugar

The Rho GTPase-mediated signaling deduced from kinome profiling could lead to the induction of the *6-sft* promoter. Alternatively it could be involved in transducing the sugar signal to other effectors. To investigate which of these possibilities is taking place, we studied the effects of inhibitors of Rho small GTPases and PI3-kinases on sucrose-mediated *6-sft* promoter activation in Arabidopsis.

**Figure 4:**
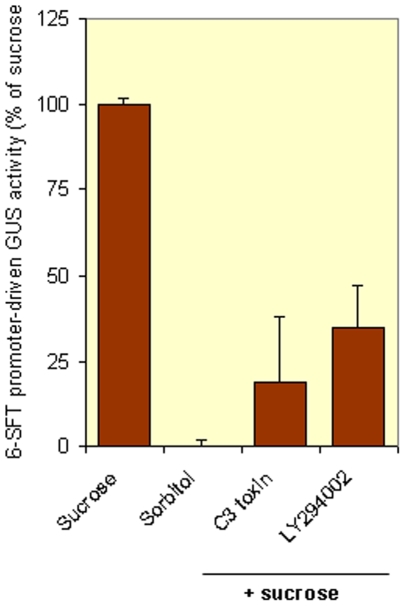
Small GTPases and PI3 kinases drive the *in planta* induction of the *6-sft* promoter. To study sugar signal transduction in *A. thaliana*, this plant was transformed with a construct in which the GUS reporter was driven by the barley *6-sft* promoter. GUS activity measured after overnight treatment of *A. thaliana* seedlings with an osmotic control is put to zero and after sucrose treatment GUS activity is put to 100%. A specific small GTPase ADP-ribosylating toxin (C3 oxin) or an inhibitor of phosphoinositide-3-OH-kinase impair this reponse to sugar.

*Clostridium dificile* toxin C3 is an inhibitor of small GTPases of the Rho family. The toxin was previously used in plants to study involvement on Rho GTPases in nod-factor signaling [76]. Application of the inhibitor at low concentrations to Arabidopsis abolished sucrose-mediated GUS induction from the *6-sft* promoter (Figure 4). This apparent need for Rho GTPases in the sugar-mediated induction of the *6-sft* promoter links kinase activities observed by kinome profiling to the induction of fructosyltransferases by sugar.

LY294002 is an inhibitor of phosphatidylinositol 3-kinase (PI3-kinase) [77], a kinase active in lipid mediated signaling. The inhibitor was used before in Arabidopsis to successfully inhibit PI3K-mediated signaling [78]. Application of LY294002 to Arabidopsis *6-sft* promoter-GUS lines does inhibit sucrose-mediated GUS induction to 35% (Figure 4), thus PI3K seems involved in the sucrose-mediated induction of the fructosyl transferase promoter. This observation suggests that phosphoinositide signaling can also in this case be linked to GTPase signaling.

Both inhibitor studies corroborate involvement of Rho GTPases and PI3-kinase in sugar signaling, as was predicted from the phosphorylation profiles seen on the PepChip. From earlier inhibitor studies tyrosine kinases ware predicted to play a role, just as could be deduced from the kinome profiling. Taken together, we provide evidence both from kinome profiling as well as inhibitor studies that Tyr-kinases, Rho GTPases and PI3-kinase are involved in sugar signal transduction towards activation of the *6-sft* promoter. As can be deduced from the kinome profiling as well as from literature these kinases possibly work together in transducing the sugar signal.

### Delineation of cell signaling pathways influenced by sugar stimulation

**Figure 5:**
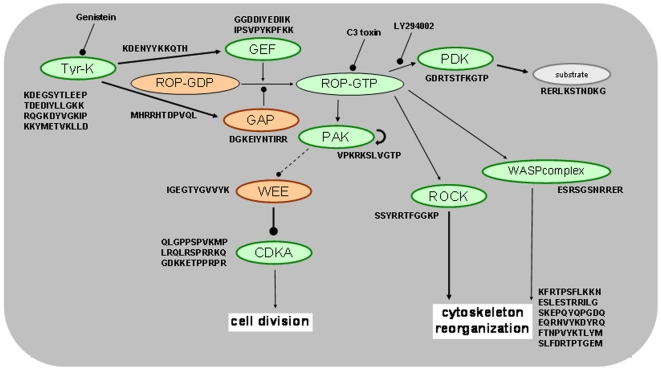
Hypothetical signal transduction scheme for sugar-induced signaling in *A. thaliana* based on the analogy with human signal transduction as well use of established inhibitors. The peptides on which elements are added to this scheme are indicated at the appropriate places (see also table 1). Green elements are up-regulated by sugar, orange-red ones are down-regulated. The results infer a central role for Ras-like GTPases (ROPs) in sugar signaling in plants.

The results obtained with both the inhibitor studies, looking at the effects certain inhibitors exert on sugar-dependent transactivation of the *6-sft* promoter as well as the results obtained by the PepChip kinome analysis were employed to construct provisional signal transduction schemes showing the differences in cellular signaling between sorbitol and sugar-stimulated plants (Figure 5). Multiple kinase activities are influenced and the results suggest a central role for small GTPases in the cellular changes evoked by sugar stimulation. Stimulation of GTPases is revealed by enhanced phosphorylation of motifs associated in GEFs with increased GTP-loading (Peptide GGDDIYEDIIK and IPSVPYKPFKK), whereas reduced hydrolysis of GTP loaded on such small GTPases would be consistent with the observed increased phosphorylation of GAP-peptide DGKEIYNTIRR. Further, a substantial number of peptides expected to indicate enhanced activity of small GTPases, display enhanced phosphorylation as well (peptide VPKRKSLVGTP, GDRTSTFKGTP, SSYRRTFGGKP, ESRSGSNRRER, DGKEIYNTIRR, EQRNVYKDYRQ, FTNPVYKTLYM). In other eukaryotes small GTPases, especially those of the Rho family, are essential for proper sugar sensing [79], and regulate a variety of biochemical pathways, including cell division, protein synthesis (via phosphatidylinositolphosphate metabolism) and cytoskeletal reorganization. The nature of the substrates of which the phosphorylation is observed suggests that this variety of functions is exerted by plant small GTPases as well (see Figure 5). Thus, from our investigations we suppose that small GTPases are central signal transduction hubs in plant sugar signaling.

Not all effects observed in this study can be directly linked to the activation of small GTPases, at least not with the knowledge on plant signaling available today. The mechanisms mediating the enhanced casein kinase activity remain unclear, but most likely share a common upstream sugar sensor with activation of small GTPases. The analogy with the animal insulin receptor on one hand and the sensitivity of transactivation of the fructosyltransferase *6-sft* promoter for genistein (a tyrosine kinase inhibitor) suggests that an upstream tyrosine kinase activity is somehow involved. But in the absence of a clear candidate gene products in the Arabidopsis genome which might act as such an upstream tyrosine kinase activator, other possibilities should be kept in mind.

Disregarding the many uncertainties that still surround sugar signaling in plants, the present study reveals that this signaling involves an intricate web of interacting mediators and provides a tantalizing glimpse as what these mediators might be. It is interesting to note that with respect to glucose signal transduction in plants a fairly large number of genetic mutants has been described that influence sugar responses (for a list, see ref [65]) and whose function is not disconcordant with the hypothetical signaling scheme to come forward from our peptide array analysis, and our data should be viewed in the context of the known body of literature with respect to glucose signaling. Especially small GTPases seem to emerge as essential signal transduction hubs coordinating the complicated biochemical changes following sugar signaling. Further studies are essential to exactly identify the gene products involved.

## Materials and Methods

### Barley and *Arabidopsis* plants

Barley (*Hordeum vulgare* L. cv. Lyric) seeds were soaked for 24 hours in running tap water and subsequenctly planted in a commercial soil mixture (UFA Haus und Garten, Bern, Switzerland). They were grown for 7 days in a growth chamber with 16 h light at 26°C and 8 h dark at 20°C. Before use they were kept 24 hours in the dark.

*Arabidopsis* seeds were sown on half-strength Murashige and Skoog medium without sucrose solidified with 1% (w/v) agar. The agar plates were incubated in a daily cycle of 10 h of light at 22°C and 14 h of darkness at 18°C. After 2 weeks of growth the seedlings were kept in the dark for 20–24 h and subjected to the different treatments.

### Cloning of the promoter

The plasmid pP6SFT3301 was constructed by replacing the 35S promoter region of the binary vector pCAMBIA3301 with approx. 1.5 kb of the barley 6-SFT promoter region (AJ306962)[7]. In this way the 6-SFT promoter controls the expression of the GUS reporter gene. The T-DNA region also has the bar gene which confers resistance to glufosinate (Basta) and enables selection of plants.

The pP6SFT3301 plasmid was introduced into *Agrobacterium tumefaciens* and Arabidopsis plants of the Col-0 ecotype were transformed using the floral dip method [80].

### Inhibitor incubation and real-time PCR of barley

5 cm of the middle part of the primary leaf was cut in two similar sized pieces. The leave pieces were submerged in 900 µl of the inhibitor solutions and subjected to vacuum (98 kPa) for 30 s. Then the leaves were half pulled out of the solution to enable a transpiration stream and kept for two hours in the dark. Sugar was added to a final concentration of 100 mM and the samples were incubated for another 24 hours in the dark at 20°C. After treatments the leaflets were frozen in liquid nitrogen and stored at −80°C.

Total RNA was extracted from the frozen leaflets of barley and treated with DNAse using the NucleoSpin RNA Plant kit (Macherey-Nagel, Oensingen, Switzerland). One microgram of RNA was reverse transcribed using AMV reverse transcriptase, AMV RT buffer (Promega, Madison, USA) and oligo(dT). The mixture was let at room temperature for ten minutes, heated up to 42°C for 15 minutes and to 56°C for two minutes. Boiling for five minutes stopped the reaction. For quantitative real time PCR the solution was diluted with water 1∶4.

Real time PCR was performed with a Gene Amp 5700 Sequence Detection System (Applied Biosystems, CA, U.S.A). The primer used to quantify the 6-SFT transcripts and the 6-SFT plasmid standard were 6-SFT (forward): 5′-TCC AAT GAG GAC GAT GGC ATG T-3′ and 6-SFT (reverse): 5′-AAT GCA TGC AAG CGA GGT-3′. The sequences of the primers of the putative histone were: (forward) 5′-CGC AAG TAC CAG AAG AGC AC-3′ and (reverse) 5′-ATG ATG GTC ACA CGC TTG GC-3′. The thermal profile was, 1 cycle 2 min at 50°C, 1 cycle 10 min at 95°C, 40 cycles 15 s at 95°C, 58°C 15 s and 1 min at 60°C. A 25 µL reaction volume consisted of 12.5 µL SYBR Green PCR master mix (Applied Biosystems), 8.5 µL water, 1.5 µL of 2.5 µM gene specific forward primers, 1.5 µL of 2.5 µM gene specific backward primers and 1 µL of diluted cDNA. Copy numbers were calculated from amplification plots of known standards for the putative histone and the 6-SFT gene. Transcript levels of a putative histone gene were used to normalize the amount of copies of 6-SFT.

The primers for the putative histone were designed after the barley EST HC11F01w found on the barley EST library “CR-EST: The IPK Crop EST Database” (website: http://pgrc.ipk-gatersleben.de/cr-est/index.php). The sequence of this barley EST was chosen because it showed the highest similarity (85%) to the coding sequence of the constitutively expressed Arabidopsis histone gene H3G (At4g40040). Real time PCR analysis showed that the barley EST HC11F01w transcript levels did not change after sugar treatments of the plants (data not shown).

### Inhibitor incubation and GUS quantification of *Arabidopsis*

Inhibitors were vacuum-infiltrated (at 20 inch Hg) for 10 min in *in vitro* grown Arabidopsis seedlings of 2 weeks old and left for 1 hour prior to sugar feeding. 100 mM of the different sugars was fed overnight in the continuing presence of inhibitors and GUS quantification was performed next morning. For the quantitative GUS assay 10 transgenic Arabidopsis seedlings were subjected to a 4-methylumbelliferyl-ß-D-glucuronide (MUG) assay. The seedlings were homogenized in 150 µl extraction buffer (50 mM Na_2_PO_4_ pH 7.0, 10 mM DTT, 1 mM EDTA, 0.1% SDS and 0.1% triton X-100). The homogenized tissue was centrifuged for 5 minutes at maximum speed and 27 µl of the supernatant was put in triplicate in a 96 well plate for measuring fluorescence. 3 µl of 10 times concentrated MUG solution (35 mg MUG per 10 µl extraction buffer) was added. The 96 well plate was sealed, shaken to mix the contents and incubated 3 h at 37°C in the dark. To stop the reaction 250 µl of a 0.2 M Na_2_CO_3_ solution was added. The fluorescence was measured using the Fluostar Optima (BMG Labtechnologies) with an excitation of 355 nm and an emission of 460 nm.

### PP2A activity

Two weeks old *Arabidopsis* plants were incubated with the appropriate sugar for 1 hour. Cell extracts were made in cell lysis buffer (Cell-Signaling Technology) with added Prefabloc (Merck). PP2A was immuun precipitated overnight at 4°C, using an antibody against the mammalian PP2A A subunit, catalytic site (#610555, Signal Transduction Laboratories) (REF). Activity was determined using the Malachite Green Phosphatase assay (Upstate) according to the protocol of the manufacturer. The amount of PP2A was quantified using Western blotting with the mammalian PP2A antibody.

### PepChip

Two weeks old *Arabidopsis* plants were incubated with the appropriate sugar for 1 hour. Cell extracts were made in cell lysis buffer according to the protocol of the manufacturer (PepScan, The Netherlands). Pepchip slides of both the Kinase1 and kinomics design were employed (the former slides exhibiting two sets of 1176 substrates selected for their broad coverage of different phosphorylation motifs and the latter contains three sets of 1040 peptide substrates based on the motifs available in human protein reference database [http://www.hprd.org/] selected for their role in signal transduction. Extensive documentation for each peptide and its source protein can be found on www.pepscan.nl/). Activation mix and γ-^33^P- ATP (Amersham Bioscience) were added according to protocol and PepChip slides were incubated at 100% humidity and 30°C for 2 hours, washed, scanned and analyzed as described before [25]. Spot intensities are normalized per PepChip and the three identical peptide sets spotted per PepChip are averaged. Three independent experiments in which one batch of plants was treated with sorbitol (100 mM) and another batch with sucrose (100 mM) for 1 hour, were analyzed on PepChips. Phosphorylation differences were determined using a heteroscedastic two-tailed Student's *t*-test on the three paired samples.

## Supporting Information

Table S1 Table with supplementary information
(0.03 MB XLS)
